# Transposition des gros vaisseaux associée aux communications interventriculaire et interauriculaire: à propos d'un cas et revue de la littérature

**DOI:** 10.11604/pamj.2013.15.24.2746

**Published:** 2013-05-13

**Authors:** Augustin Mulangu Mutombo, Olivier Mukuku, Toni Kasole Lubala, Maguy Sangaji Kabuya, Paul Makinko Ilunga, Marcellin Bugeme, Oscar Numbi Luboya

**Affiliations:** 1 Faculté de Médecine, Université de Lubumbashi, RD Congo

**Keywords:** Transposition des gros vaisseaux, Communication interventriculaire, Communication interauriculaire, Malformation cardiaque, Transposition of the great vessels, interventricular communication, interatrial communication, heart defect

## Abstract

Nous rapportons une observation d'un nourrisson de 5 mois présentant une transposition des gros vaisseaux associée aux communications interventriculaire et interauriculaire. Il est né à terme sans aucun facteur de risque retrouvé dans les antécédents maternels. Le diagnostic est posé, grâce à une échocardiographie, à 5 mois après sa naissance lors de la survenue d'une cyanose et d'un malaise anoxique. Une prise en charge symptomatique a permis de stabiliser l’état du patient mais suite à l'absence d'un traitement chirurgical, il est décédé à domicile 3 semaines après sa sortie de l'hôpital. Dans les pays en développement, le diagnostic de la transposition des gros vaisseaux est souvent fait en période postnatale et son pronostic reste fatal par manque des centres médico-chirurgicaux spécialisés.

## Introduction

La transposition des gros vaisseaux (TGV) est une malformation cardiaque congénitale fréquente, caractérisée par une inversion des gros vaisseaux de la base du c'ur (aorte et artère pulmonaire). Incompatible avec la vie en l'absence de traitement chirurgical, son pronostic a été transformé par le développement de la chirurgie cardiaque néonatale [1].

Bien que l’évolution de l′imagerie médicale soit aujourd'hui capable de révéler cette anomalie cardiaque en anténatale, nous notons malheureusement que dans certaines régions ce diagnostic est souvent de révélation tardive si bien qu'il donne l'impression d’être beaucoup plus rare et hypothéquant ainsi l'avenir du jeune nourrisson.

Nous rapportons ici un cas de transposition des gros vaisseaux avec communications interventriculaire (CIV) et interauriculaire (CIA) vicariantes sans sténose pulmonaire chez un nourrisson de 5 mois avec un bon état nutritionnel hospitalisé en novembre 2009 aux Cliniques Universitaires de Lubumbashi en République Démocratique du Congo. Signalons qu′il s′agit ici du premier cas rapporté dans la littérature où une communication interauriculaire est associée à une communication interventriculaire dans une TGV.

## Patient et observation

Un enfant âgé de 5 mois de sexe masculin est admis aux urgences pédiatriques des Cliniques Universitaires de Lubumbashi pour difficultés respiratoires sévères dans un contexte afébrile.

A l'anamnèse, les parents de niveau socio-économique précaire décrivent la difficulté respiratoire d'allure progressive. L'enfant est cadet d'une fratrie de 4 enfants tous en vie et les deux parents sont également en vie et en bonne santé apparente. Sa mère signale qu'elle n'avait pas suivi les consultations prénatales. Notons que le patient était né eutociquement avec un poids 3400 grammes sans malformations visibles et n'a jamais été hospitalisé depuis la naissance. Les antécédents maternels n'ont révélé aucune particularité.

A l'examen clinique, le patient est dyspnéique. Il a un poids de 7 kg et une taille de 69cm. Son état nutritionnel est satisfaisant (Z-score poids/âge est de -0.629 et Z-score taille/âge est de 1,473). Il présente des battements des ailes du nez, un tirage sous-sternal, une tachycardie à138 battements/min et une tachypnée à 75 cycles/min. A l'auscultation, nous n'avons pas perçu des bruits surajoutés. Un diagnostic de broncho-pneupathie aigue dyspnéisante est posé, un traitement fait de kinésithérapie respiratoire, de l'oxygénothérapie et la déxaméthasone en IV est administrée à la dose de 0.5mg/Kg.

**Figure 1:**
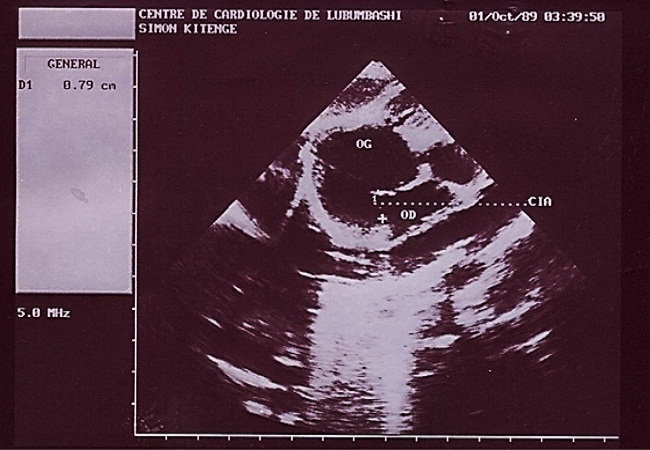
Image échographique montrant les deux oreillettes (OG et OD) et leur communication (CIA)

**Figure 2:**
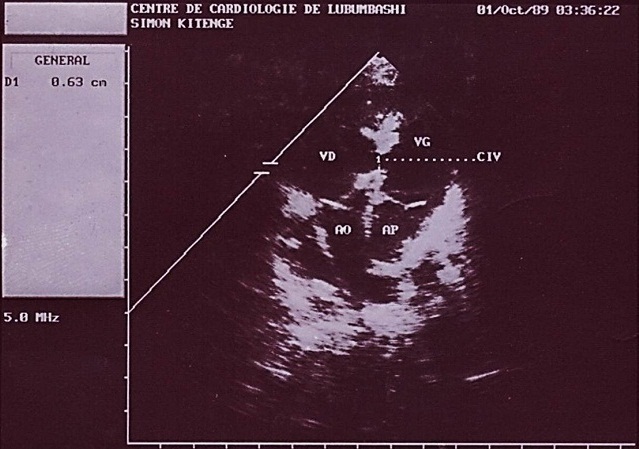
Image échographique montrant les deux ventricules (VG et VD) et leur communication (CIV)

**Figure 3:**
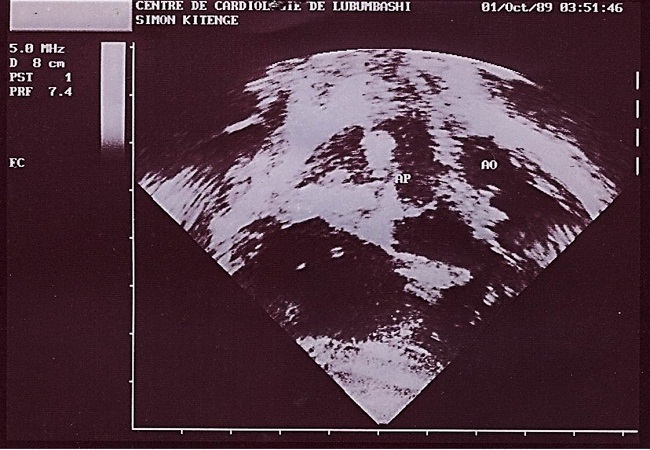
Image échographique montrant l'aorte (AO) et l'artère pulmonaire (AP)

**Figure 4:**
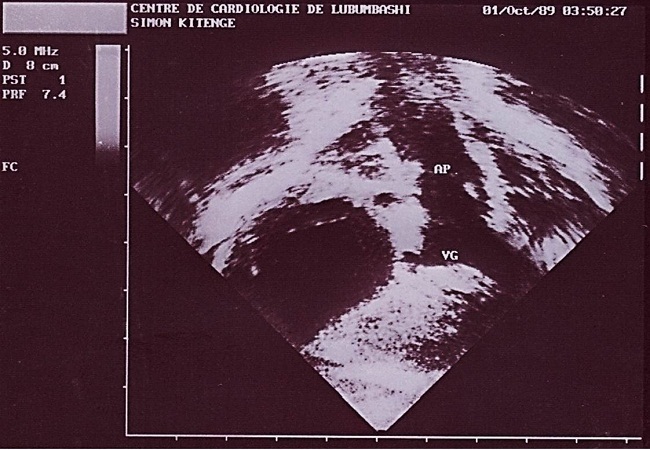
Image échographique montrant l'artère pulmonaire (AP) prenant son origine dans le ventricule gauche (VG)

**Figure 5:**
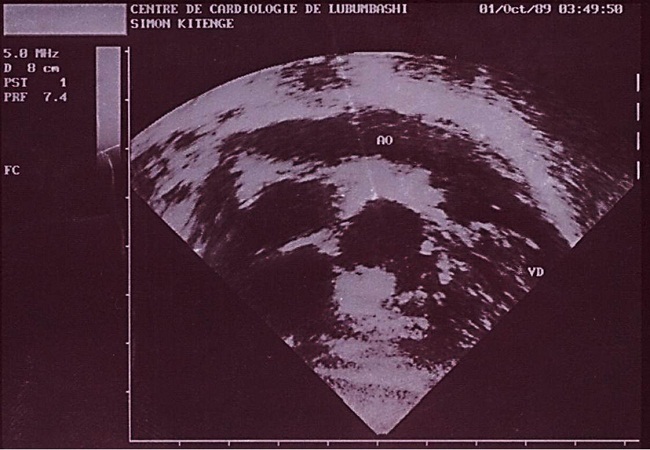
Image échographique montrant l'aorte (AO) prenant son origine dans le ventricule droit (VD)

Au deuxième jour de son hospitalisation apparait une agitation, des pleurs incessants, une cyanose palmoplantaire et buccale ainsi que des convulsions tonico-cloniques. Le diagnostic de malaise anoxique sur cardiopathie congénitale cyanogène est retenu et l'enfant bénéficiera du diazépam 1 mg/kg en intra rectale, du sérum glucosé 5%, du bicarbonate 2 mEq/kg et de l'oxygénothérapie. La radiographie du thorax faite révèle une cardiomégalie avec l'indice cardiothoracique à 0.64 et l’échocardiographie faite en urgence montre une transposition des gros vaisseaux avec communications interventriculaire (large de 6 mm) et interauriculaire (large de 8 mm) vicariantes sans sténose pulmonaire (Figure 1, Figure 2, Figure 3, Figure 4, Figure 5). En dehors de celles-ci, les autres structures cardiaques sont normales.

Au dixième jour, l’état clinique est stable et l'enfant est autorisé de quitter l'hôpital. Vingt jours plus tard, le patient meurt à domicile dans un tableau clinique identique à celui de son admission à l'hôpital par manque de traitement chirurgical que nous ne sommes pas en mesure de dispenser dans notre milieu.

## Discussion

La transposition des gros vaisseaux est une malformation cardiaque congénitale qui fut décrite pour la première fois par Mathew Baillie en 1797, mais le terme de transposition a été seulement appliqué en 1814 par Farre, signifiant que l′aorte et le tronc pulmonaire ont été placés à travers la cloison ventriculaire. Elle représente 5 à 7% de toutes les cardiopathies congénitales, correspondant à une incidence de 20 à 30 par 100000 naissances vivantes [2, 3]. Dans une étude menée à Malte, Grech rapporte 3% des cas de transpositions des gros vaisseaux dans l'ensemble de cardiopathies congénitales enregistrées entre 1990 et 1994 [4].

Comme pour la plupart de malformations cardiaques congénitales, la prédominance masculine est à noter dans la TGV avec un sexe ratio M/F variant, dans la littérature, de 1.50/1 à 3.22/1 [5–7].

La TGV est souvent associée à d'autres malformations cardiaques et dans environ 10% des cas, elle est accompagnée d′autres malformations extracardiaques [8]. S'agissant de notre patient, il est de sexe masculin et aucune malformation extracardiaque n'avait été retrouvée. Les malformations cardiaques associées sont la CIV et la CIA. Cette double communication associée à une TGV est très rarement observée et dans la littérature parcourue nous n'avons pas trouvé des cas similaires.

Son étiologie demeure jusqu’à présent inconnue mais certains facteurs de risque ont été retrouvés à savoir l′exposition maternelle à des rodenticides et des herbicides, l′utilisation maternelle de médicaments antiépileptiques ainsi que le diabète gestationnel [2]. Il faudra souligner que, grâce à des avancées significatives en matière de la génétique, dans cette cardiopathie longtemps considérée comme typiquement sporadique et «; accidentelle »;, l'implication de trois gènes (ZIC3, CFC1 et PROSIT240) a été mise en évidence mais la responsabilité du facteur génétique n'a été prouvée que dans une minorité des cas [9, 10]. Dans notre observation, nous n'avons relevé aucun facteur de risque et faute des moyens financiers aucun examen paraclinique n'a été réalisé sur le plan génétique.

Lorsqu'associée à une CIV comme dans notre observation, la clinique de la TGV est souvent muette suite au mélange satisfaisant entre les deux circulations (artérielle et veineuse). La cyanose passe inaperçue et n'apparait qu'au cours des épisodes de pleurs, cris ou d'agitation et dans ce cas, apparaissent les signes d'insuffisance cardiaque congestive en raison du travail excessif ventriculaire. Ainsi, le patient présente une tachycardie, une tachypnée, un diaphorèse et parfois un bruit de galop; et tardivement, une hépatomégalie et une prise de poids faible sont à noter [2]. Ces formes associées à une CIV sont souvent mieux tolérées à la naissance comparativement aux formes simples dans la mesure où cette CIV, surtout si elle est large, ne se ferme pas aussi rapidement et permet un meilleur mélange sanguin, mais au prix d′une insuffisance cardiaque.

Le diagnostic de certitude peut être obtenu en prénatal comme en postnatal et est donné par l′échographie cardiaque. Quand elle est faite en période prénatale, elle permet d'améliorer la première gestion néonatale et réduire la morbidité et la mortalité surtout dans les pays occidentaux comme c'est le cas, actuellement, de la France où le diagnostic prénatal de la TGV est fait dans 72.5 à 80% des cas [1, 11] et le taux de mortalité périnatale dû à cette maladie a sensiblement baissé passant de 23.5% entre 1983 et 1988 à 5% entre 1995 et 2000 [11]. Le fait du manque de suivi des consultations prénatales au cours de la grossesse explique le diagnostic de cette maladie en période postnatale dans notre observation. D'où l'importance de sensibiliser les femmes enceintes dans les pays à faible pénétration sanitaire comme la République Démocratique du Congo à suivre les consultations prénatales car un diagnostic prénatal permettrait d'envisager l'option de poursuivre ou d'interrompre la grossesse.

Le traitement de cette pathologie ne consiste qu'en une correction chirurgicale (réparation anatomique) et avec l′apparition de techniques chirurgicales plus récentes et améliorées, la survie à long terme est approximativement 90% à 15 ans [12]; la mortalité après cure chirurgicale en cas de TGV associée à une CIV est de 5.5% [13].

Par manque de centres de chirurgie spécialisés permettant de prendre en charge ces malformations cardiaques dans notre milieu comme pour la plupart de pays en développement, nous recourons à une prise en charge symptomatique comprenant selon les cas une ventilation assistée, la mise en place d'une voie d'abord veineuse de bonne qualité, une correction des troubles ioniques, acido-basiques et hémodynamiques avec surveillance en unité de soins intensifs mais aussi à un traitement palliatif fait des prostaglandines E1 en intraveineuse.

La survie post-natale n'est possible que grâce à l'existence de shunt intracardiaque (CIV, CIA) ou extracardiaque (canal artériel); cependant, en l'absence de traitement chirurgical, le pronostic est extrêmement sombre (moins de 5% des patients atteignent l’âge de 1 an) [1].

## Conclusion

La transposition des gros vaisseaux est une malformation cardiaque congénitale grave, qui en l'absence de traitement approprié, conduit au décès de l'enfant. Elle est pratiquement et constamment létale dans les pays en développement qui ne sont pas équipées dans le domaine de la chirurgie cardiaque néonatale, mais présentant un excellent pronostic dans les pays développés où l'on trouve des centres médico-chirurgicaux hautement spécialisés. D'où la nécessité d'un diagnostic anténatal permettant d'envisager une interruption de la grossesse.
